# Development and Pilot Testing of Standardized Food Images for Studying Eating Behaviors in Children

**DOI:** 10.3389/fpsyg.2020.01729

**Published:** 2020-07-21

**Authors:** Samantha M. R. Kling, Alaina L. Pearce, Marissa L. Reynolds, Hugh Garavan, Charles F. Geier, Barbara J. Rolls, Emma J. Rose, Stephen J. Wilson, Kathleen L. Keller

**Affiliations:** ^1^Metabolic Kitchen and Children’s Eating Behavior Laboratory, Department of Nutritional Sciences, The Pennsylvania State University, State College, PA, United States; ^2^Evaluation Sciences Unit, Division of Primary Care and Population Health, Department of Medicine, School of Medicine, Stanford University, Stanford, CA, United States; ^3^Department of Psychiatry, University of Vermont Medical School, Burlington, VT, United States; ^4^Department of Psychological Sciences, University of Vermont Medical School, Burlington, VT, United States; ^5^Laboratory, Department of Human Development and Family Studies, The Pennsylvania State University, State College, PA, United States; ^6^Laboratory for the Study of Human Ingestive Behavior, Department of Nutritional Sciences, The Pennsylvania State University, State College, PA, United States; ^7^Laboratory, Prevention Research Center, The Pennsylvania State University, State College, PA, United States; ^8^Addiction Smoking and Health Laboratory, Department of Psychology, The Pennsylvania State University, State College, PA, United States; ^9^Metabolic Kitchen and Children’s Eating Behavior Laboratory, Department of Food Sciences, The Pennsylvania State University, State College, PA, United States

**Keywords:** standardized food images, food pictures, portion size, energy density, food cues, children, eating behaviors

## Abstract

Food images are routinely used to investigate the cognitive and neurobiological mechanisms of eating behaviors, but there is a lack of standardized image sets for use in children, which limits cross-study comparisons. To address this gap, we developed a set of age-appropriate images that included 30 high-energy-dense (ED) foods (>2.00 kcal/g), 30 low-ED foods (<1.75 kcal/g), and 30 office supplies photographed in two amounts (i.e., “larger” and “smaller”). Preliminary testing was conducted with children (6–10 years) to assess recognition, emotional valence (1 = very sad, 5 = very happy), and excitability (1 = very bored, 5 = very excited). After the initial testing, 10 images with low recognition were replaced; thus, differences between Image Set 1 and Image Set 2 were analyzed. Thirty (*n* = 30, mean age 8.3 ± 1.2 years) children rated Set 1, and a different cohort of 29 children (mean age 8.1 ± 1.1 years) rated Set 2. Changes made between image sets improved recognition of low-ED foods (Set 1 = 88.3 ± 10.5% vs. Set 2 = 95.6 ± 10.6%; *p* < 0.0001) and office supplies (83.7 ± 10.5 vs. 93.0 ± 10.6%; *p* < 0.0001). For the revised image set, children recognized more high-ED foods (98.4 ± 10.6%) than low-ED foods (95.6 ± 10.6%; *p* < 0.05) and office supplies (93.0 ± 10.6%; *p* < 0.0001). Recognition also improved with age (*p* < 0.001). Excitability and emotional valence scores were greater for high-ED foods compared with both low-ED foods and office supplies (*p* < 0.0001 for both). However, child fullness ratings influenced the relationship between excitability/emotional valence and category of item (*p* < 0.002). At the lowest fullness level, high-ED foods were rated the highest in both excitability and emotional valence, followed by low-ED foods and then office supplies. At the highest fullness level, high-ED foods remained the highest in excitability and emotional valence, but ratings for low-ED foods and office supplies were not different. This suggests that low-ED foods were more exciting and emotionally salient (relative to office supplies) when children were hungry. Ratings of recognition, excitability, and emotional valence did not differ by image amount. This new, freely available, image set showed high recognition and expected differences between image category for emotional valence and excitability. When investigating children’s responsiveness to food cues, specifically energy density, it is essential for investigators to account for potential influences of child age and satiety level.

## Introduction

Food images have become standard research tools for investigating the cognitive processes associated with eating behaviors (Fedoroff et al., 1997; Nederkoorn and Jansen, 2002; Lawrence et al., 2012; Kemps et al., 2016). When compared with exposure to actual foods, tastes, or smells, food images produce reliable neurological responses (van der Laan et al., 2011) that predict subsequent eating behaviors (Boswell and Kober, 2016). Responses to food images also show ecological validity, in that they vary with physiological state (LaBar et al., 2001; Seibt et al., 2006; Kroemer et al., 2013) and by clinical characteristics, such as dietary restraint (Blechert et al., 2010; Burger and Stice, 2011; Kemps et al., 2016) disordered eating (Blechert et al., 2011) and obesity (Martin et al., 2010; Stoeckel et al., 2008). Moreover, although exposing participants to actual foods may not be feasible in some settings (e.g., functional magnetic resonance imaging) and may introduce variability as food quality and freshness change over time, food images offer a practical and flexible alternative for studying human eating behaviors that can be standardized across experimental settings. Although there are several publicly available databases of food images developed for use with adults (Blechert et al., 2014, 2019; Foroni et al., 2013; Miccoli et al., 2014; Charbonnier et al., 2016; King et al., 2018) few have been tested for appropriateness with children (Charbonnier et al., 2016). Therefore, the aim of this study was to develop and conduct pilot testing on a set of high-quality food and non-food control images appropriate for studying eating behaviors in pediatric samples.

Although the development of standardized image sets has been promoted to improve cross-study replicability (Smeets et al., 2012; Foroni et al., 2013; Blechert et al., 2014; Charbonnier et al., 2016) the wide variations of food culture and cuisine across geographic and demographic settings make it unlikely that one image set will suit the needs of every investigator. Therefore, image sets developed and validated primarily for adults may not be ideal for use with children. One of the most extensive open image databases (Food-pics) includes 896 food images varying in food type and cultural origin (Blechert et al., 2014, 2019). However, compared with adults, adolescents tested with these images showed lower recognition, and they misclassified the calorie content for over 20% of the foods (Jensen et al., 2016). Charbonnier et al. (2016) developed another freely available, standardized image set that has been tested with children from several European countries. However, this set also showed lower recognition among children compared with adults, which suggests need to create or modify image sets for pediatric cohorts. Further, both Food-pics (Blechert et al., 2014, 2019) and the image set created by Charbonnier et al. (2016) were developed in Europe, and thus the images of foods in the set may be unfamiliar or eaten less frequently among children from the United States (e.g., chocolate éclairs, Kinder Bueno, and sausages). Therefore, the development of a set of images tailored to the dietary customs of US children would complement the currently available databases and help to advance the field.

A gap in the literature with respect to most prior food image sets is the standardization of food portion size. Because many of the prior image sets were developed for use in neuroimaging studies, the size of the food image is often quantified as the number of non-white pixels (i.e., food image) relative to total pixels (i.e., plate and background) (Foroni et al., 2013; Blechert et al., 2014, 2019). This produces ideal results with respect to standardization across images, but it results in unrealistic serving sizes for many foods, particularly when comparing low-energy-dense (ED) (e.g., plate of fresh fruits or vegetables) with high-ED foods (e.g., plate of chocolate candies or cake). This effect is exacerbated in children, who typically eat smaller portion sizes of many foods than adults (Fox et al., 2006). While Brunstrom and Rogers (2009) have developed a food image dataset with extensive options for portion size, the foods contained in this catalog are targeted primarily at a European population. Additionally, portion sizes for many foods vary by country; thus, adjustments would be needed in both food type and serving size when using these images with US children.

It is well established that large portions of high-ED foods promote excess energy consumption among both children (Fisher et al., 2003, 2007; Mathias et al., 2012; Kling et al., 2016; Smethers et al., 2019) and adults (Kral and Rolls, 2004; Rolls et al., 2004, 2007). However, the cognitive mechanisms underlying the relationships between portion size, energy density, and energy intake have not been determined. To address this gap, we aimed to develop a set of high-quality food images appropriate for use with children to investigate the neurocognitive mechanisms underlying eating behaviors. We developed images that varied in portion size (larger food vs. smaller food) and ED (high-ED foods > 2 kcal/g vs. low-ED foods < 1.75 kcal/g), along with non-food objects that also varied in amount (large vs. small quantities). We conducted two pilot tests to assess 6- to 10-year-old children’s familiarity (i.e., recognition), excitability, and emotional valence in response to the images. In the pilot tests, we had several hypotheses. First, because we purposely designed the image sets to include familiar foods and objects, we hypothesized that recognition for high-ED foods, low-ED foods, and office supplies would be similar. Second, we hypothesized that children would rate high-ED foods as more exciting and emotionally positive (i.e., happier) than low-ED foods and office supplies. Finally, we hypothesized that there would be no difference in recognition, excitability, and emotional valence between the images of larger and smaller amounts (i.e., portions).

## Materials and Methods

### Study Design

A set of 180 images that was matched on multiple image properties was developed in two stages. Initial pilot testing of thirty 6- to 10-year-old children indicated the need to revise 10 of the images, and the revised image set was tested in a separate cohort of twenty-nine 6- to 10-year-old children. Thus, there were two versions of the image sets: Image Set 1 and Image Set 2. Both versions included 30 high-ED foods, 30 low-ED foods, and 30 office supplies photographed in two amounts (i.e., large or small quantities). To be consistent with the evaluations collected on the International Affective Picture System (IAPS) (Lang et al., 2008) a standardized image set created by the National Institutes of Mental Health, children rated the recognition, emotional valence, and excitability for each image by using a computer-administered survey completed during laboratory visits. The IAPS images are some of the most frequently used for studying emotional and cognitive processing in children, and there are normative cutoffs for the rating attributes. Collecting data on these attributes in the current study will therefore allow for broader cross-study comparisons. Differences between Image Sets 1 and 2, and between image category (high-ED foods, low-ED foods, or office supplies), and amount (large or small quantities) were tested. Secondary analyses were conducted to further investigate how these rated characteristics differed between savory and sweet foods.

### Photographing Protocol

All images were photographed by a local professional photographer with experience in producing food images for bakeries. The images were photographed with a Nikon d750 (Ayutthaya, Thailand) camera (with 24.3 million effective pixels, 100-12,800 ISO; sensitivity of the image sensor), Expeed 4 Image Processor, 51 autofocus (AF) points, group area AF, and full frame (FX; 26X24) image area of 6,016 × 4,016 [L], 4,512 × 3,008 [M], and 3,008 × 2,008 [S]. A 2.8 in. (72.1 mm) × 2.1 in. (52.4 mm) Nikon Nikkor AF-S 50 mm, f/1.8 maximum aperture lens with FX/35-mm format, minimum aperture of f/16, maximum angle view of 47°, seven diaphragm blades, and minimum focal distance of 1.48 ft (0.45 m) was used. Images were captured in Nikon Electronic Format (NEF; raw) 12- or 14-bit data with lossless compressed. For the images of high-ED foods, the ISO ranged from 200 to 250 ISO units, focal length ranged from 3.5 to 11.0 mm, and shutter speed ranged from 1/500 to 1/15 s. For images of low-ED foods, the ISO ranged from 200 to 640 units, focal length ranged from 4.0 to 11.0 mm, and the shutter speed ranged from 1/400 to 1/15 s. For the office supply images, the ISO ranged from 200 to 640 units, focal length ranged from 2.8 to 11.0 mm, and shutter speed ranged from 1/640 to 1/40 s.

**FIGURE 1:**
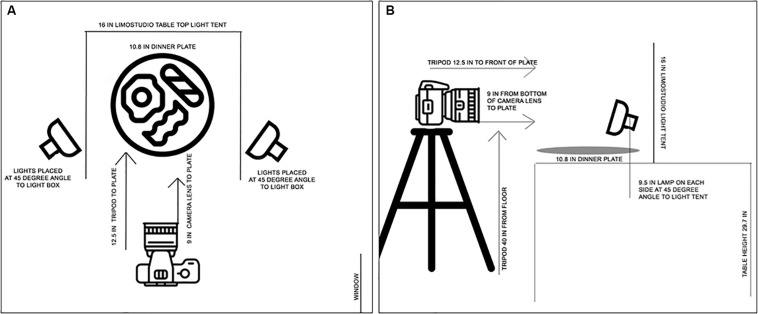
Diagram depicting placement of food or office supplies on a plate in relation to camera, tripod. lights. and light box used in photography protocol. **(A)** Shows the bird’s-eye view of the equipment and objected of interest photographed by the professional photographer. **(B)** Shows the eye level view of the equipment and object of interest photographed by the professional photographer.

The camera was mounted on a tripod and positioned in relation to the food according to the diagram in Figures 1A,B. The middle of the tripod base was 40 in. from the floor and 12.5 in. from the front edge of the plate. The plate was 9 in. from the bottom of the camera lens. The lens of the camera was parallel to the floor to create photographs that represented how a plate of food would be viewed by an average-height child when seated at a 30-in. height table. The table was covered with a gray tablecloth to create a neutral background for all foods and office items. The food was photographed on either a 10.8-in. white dinner plate (Corelle Vitrelle, Rosemont, IL, United States) or a 6.8-in. white bowl (Corelle Vitrelle, Rosemont, IL, United States), depending on which was most appropriate.

To create an optimal lighting condition, the lights in the room were turned off, and the blinds were shut to provide indirect natural light. A continuous lighting kit (i.e., LimoStudio 16-in. by 16-in. table top light tent) with two lamps (i.e., 5,500 K, 600 lumens, 120° beam angle) and maximum height of 9.5 in. was used to light the images. The lamps were placed outside of the light box, on each side, at a 45° angle. The plate or bowl was placed in the exact center (8 in. from each side) of the light tent studio.

Because the images were taken across multiple days, they were edited to account for varying amounts of natural light. Adobe Lightroom 5 and Adobe Photoshop Elements 15 software programs (San Jose, CA, United States) were used to standardize differences in contrast, brightness, and color distributions. First, the average contrast, brightness, and color distribution were analyzed by group (high-ED foods, low-ED foods, and control) and size (small, large). Images were imported as (NEF; raw) files into Adobe Lightroom 5 to “batch” edit to adjust for white balance using an automatic setting. Then, each image was manually adjusted for blue, green, and red saturation. After all color distributions were verified to be similar, images were cropped to create uniformity. The images were then exported from Lightroom with sharpening for web and 300 dots per inch (dpi) as.jpeg images to Photoshop. In Photoshop, the photographer removed spots or wrinkles on the table cloth, plate, bowl, or food item by editing or cloning. The image of the small portion of orange had the yellow and red color distributions altered in Photoshop to enhance the orange color of the food. Images with brand logos (e.g., Oreo) on the foods or recognizable numbers/text on the office supplies were cloned at 100% opacity to remove these identifying marks. Images were saved from Adobe Photoshop Elements 15 as.jpeg files with maximum image quality.

### Image Selection

As listed in Table 1, foods in the high-ED and low-ED categories were selected to represent those that were commonly consumed by children in the United States on the basis of available national data (Smiciklas-Wright et al., 2003). In selecting foods for this dataset, we chose those that would be commonly consumed in childcare centers and schools and that would be familiar to the majority of children living in central Pennsylvania. In this first iteration, we did not include ethnic foods, multi-item meals, or beverages in order to maintain a small dataset that would be able to be evaluated by 6- to 10-year-old children in a single test session. The high-ED foods ranged from 2 to 5.9 kcal/g (mean ± *SD*; 4.3 ± 1.38 kcal/g); and the low-ED foods ranged from 0.2 to 1.75 kcal/g (0.69 ± 0.54 kcal/g). Both low- and high-ED food categories were balanced for predominant taste characteristic by including 15 sweet and 15 savory foods. The selected office supplies were chosen to resemble the food images in terms of the shape of the item and the space taken up on the plate. Additionally, we included the same colors across the group of office supplies as the colors included in food images.

**TABLE 1:** Descriptions of high-ED foods, low-ED foods, and control office supplies included in two versions of a standardized image set developed to investigate the cognitive and neurobiological mechanisms of eating behaviors in 6- to 10-year-old children.

	**High-ED foods**				**Low-ED foods**			**Control**
**Food name**	**Energy density**	**Large amount (g)**	**Small amount (g)**	**Food names**	**Energy density**	**Large amount (g)**	**Small amount (g)**	**Office supply (all depicted in large and small amounts)**
**Savory foods**								
Bacon	4.60	88.5	50.5	Baby carrots	0.35	111.5	33.6	Binder clip
Bagel with cream cheese	2.70	129.7	67.6	Broccoli	0.26	46.8	26.2	Calculator
Cheeseburger	4.20	329.4	173.4	Brown rice	1.47	94.8	32.5	Chalk
Chicken nuggets	2.80	173.4	76.2	Cherry tomatoes	0.20	158.1	36.4	Coin roll
Fish sticks	2.40	106.7	53.5	Chicken soup	0.37	340.1	135.1	Compass
French fries	1.60	115.8	54.6	Cottage cheese (Version 1)^b^	0.53	231.2	465.1	Craft scissors
Grilled cheese sandwich	6.60	285.0	139.7	Cucumbers	0.15	150.3	61.1	Envelopes (Version 1)^e^
Italian submarine sandwich	7.60	223.2	109.7	Green beans	0.29	186.6	93.5	Eraser
Macaroni and cheese	2.60	342.3	29.1	Green salad	0.24	34.9	7.1	Index card
Peanut butter crackers	5.10	52.2	25.7	Mashed potato (Version 1)^c^	0.80	71.2	24.0	Marker
Peanuts, lightly salted	5.80	77.7	28.4	Peas	0.77	111.4	27.1	Notebook
Pizza, cheese	2.60	170.5	87.8	Pepper, red	0.31	87.7	42.9	Notepad
Potato chips	5.70	49.0	14.6	Potatoes, baby (Version 2)^c^	0.89	199.4	81.0	Padlock
Pretzels	3.80	63.9	32.0	Red beans and rice	1.75	257.6	76.42	Paintbrush
Tortilla chips with cheese	5.60	152.2	58.3	Tomato soup	0.46	275.2	140.6	Paints
				Turkey, grilled (Version 1)^d^	0.89	110.0	55.0	Paperclip, yellow (Version 2)^e^
				Turkey, grilled (Version 2)^d^	0.89	74.1	43.3	Paperclip green
				Turkey, deli (Version 2)^b^	0.89	141.7	57.0	Pencil full
Sweet foods								Post-it Note
Browning, fudge	5.00	111.4	55.2	Apple slices	0.52	145.9	70.7	Post-it Note tape
Cake with frosting	4.20	140.4	68.7	Applesauce	0.80	364.4	87.7	Push pin
Chocolate candies	5.10	64.1	30.8	Banana with peel	0.89	112.7	52.3	Rope
Chocolate ice cream	2.10	247.0	87.3	Blueberries	0.57	207.1	51.4	Rubber band
Chocolate peanut butter candy	4.97	115.1	15.7	Fruit cocktail, canned	0.63	204.4	80.5	Ruler
Chocolate cream pie	3.50	147.3	74.1	Cantaloupe	0.34	262.8	91.5	Sharpie brown
Chocolate sandwich cookie	5.20	80.7	34.7	Corn	0.86	210.9	90.5	Sharpie yellow
Cinnamon bun with frosting	3.30	153.1	70.4	Gelatin dessert	0.73	150.8	104.7	Stapler
Coffee cake (Version 1)^a^	3.90	41.0	20.5	Oranges	0.49	638.4	155.3	Staples
Coffee cake (Version 2)^a^	4.30	28.6	56.0	Pineapple rings	0.71	189.8	91.4	Tab divider
Doughnut, glazed	4.40	143.6	66.8	Popsicle	0.64	94.8	48.0	Thumbtack
Fruit candy chews	4.00	77.5	39.8	Red grapes	0.71	250.0	71.4	
Muffin, blueberry	3.90	200.4	100.6	Strawberries	0.32	431.7	122.8	
Rice Krispies treat	4.10	87.8	21.2	Watermelon	0.30	382.1	202.9	
Sugar cookie with sprinkles	4.60	78.5	39.8	Yogurt	0.88	451.5	109.6	
Waffle and Syrup	6.10	148.8	86.3					

**ED, energy dense. *^a^ Brand and shape of coffee cake were changed between Image Set 1 and Image Set 2. ^b^ Cottage cheese in Image Set 1 was changed to deli turkey in Image Set 2. ^c^ Mashed potatoes in Image Set 1 was changed to whole baby potatoes in Image Set 2. ^d^ Brand, shape, and number of pieces of grilled turkey were changed between Image Sets 1 and 2. ^e^ Envelopes in Image Set 1 were changed to yellow paperclips in Image Set 2.***

**FIGURE 2:**
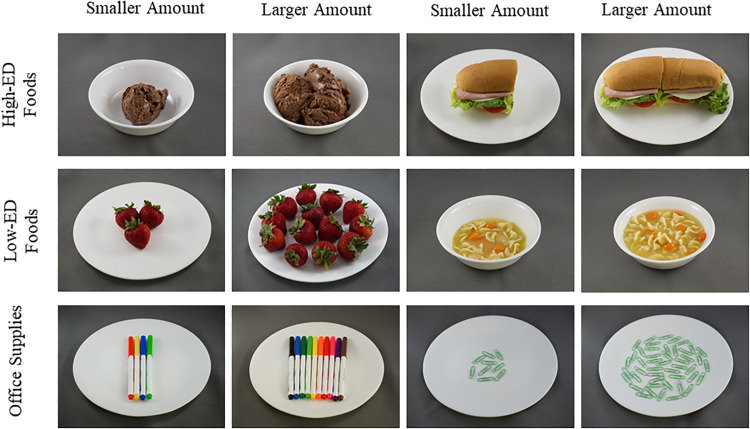
Example images of smaller and larger amounts of high-energy-dense foods. low-energy-dense foods and office supplies from a standardized image set developed investigate the cognitive and neurobiological mechanisms of eating behaviors in 6- to 10-year-old children.

For each of the foods, the small portion size was chosen as the standard portion on the Nutrition Facts Panel or by referencing the amount of that food customarily consumed by children (Smiciklas-Wright et al., 2003). For most foods, the large portion size was established by doubling the size by weight of the small portion. However, unit foods (e.g., bagels and cookies) were not broken into pieces, so the large portions for these foods were achieved by doubling the units (i.e., one cookie for small and two cookies for large). To maintain consistency in average portion size across the large and small conditions of low-ED foods, high-ED foods, and office supplies, portion sizes of some of the foods were manually adjusted during the photo shoot to establish reasonable serving sizes for 6- to 10-year-old children. Example images of small and large amounts of high-ED foods, low-ED foods, and office supplies are depicted in Figure 2. All images are available for download at https://osf.io/ynjqw/.

### Participants

Participants were recruited via flyers posted in the community; advertisements on popular internet sites, parent groups, family-oriented periodicals, and newsletters; and at schools. Parents of potential participants contacted research staff and were screened by phone or email. To be eligible for the study, children had to be 6–10 years old at enrollment and in good health on the basis of parent report. Children were excluded if they were currently taking cold or allergy medication or other medications known to influence cognitive function, taste, or appetite. Additionally, children were excluded if they had the following conditions: learning disability [e.g., attention-deficit disorder (ADD)/attention-deficit/hyperactivity disorder (ADHD), language delays, and autism], other neurological or psychological conditions (e.g., anxiety, depression, or schizophrenia), or a preexisting medical condition that would influence intake. Families were excluded if the primary parent in charge of making feeding decisions was unable to attend the study visit or if the family reported plans to move away from the area in the next year. Because this was a pilot study, a power calculation was not conducted.

### Data Collection

The participating child and the primary parent in charge of making feeding decisions attended one laboratory visit. Data collection was completed from October 2017 through February 2019, and all procedures were approved by the Institutional Review Board of The Pennsylvania State University. Parents signed informed consent to allow their children to participate, and children provided written assent.

#### Anthropometrics

Parent and child height and weight were assessed without shoes and coats on a stadiometer (Seca^®^ model 212 Chino, CA) and standard scale (Detecto^®^ model 437, Webb City, MO). Height was measured to the nearest 10th of a centimeter, and weight was measured to the nearest 10th of a pound. Parent measurements were converted to body mass index (BMI; kg/m^2^); and child measurements were converted to BMI (kg/m^2^) and BMI *z*-score on the basis of the Centers for Disease Control and Prevention growth charts as the weight-to-height ratio for age and sex (Cole et al., 2000).

#### Parental Questionnaires

During the study visit, parents completed a demographic questionnaire that included questions on child birth anthropometrics and feeding information, child and parent ethnicity and race, household characteristics, parents’ education and employment, and use of federally funded nutrition programs. These data were collected to gather information on potential characteristics that could systematically influence children’s ratings and were examined as potential covariates in analyses.

#### Child Procedures and Assessments

Prior to rating the images, children rated the fullness level using a validated, pictorial fullness scale (Keller et al., 2006). After rating fullness, children completed a computer-based questionnaire to rate attributes of each image. To avoid variability in performance due to differences in reading ability across children, a trained research assistant read each question to the child and used the computer mouse to record the child’s answer on the computer screen as the child pointed to the correct place on the scale. Images of foods (low- and high-ED foods, and small and large portions) and office supplies (small and large amounts) were presented with three questions assessing familiarity, emotional valence, and excitability. These attributes were selected for consistency with the IAPS catalog of pictures (Lang et al., 2008). Each image (16 × 11.5 cm) was presented individually on the computer screen. Familiarity of the image was assessed by asking children to name the depicted image. Excitability and emotional valence were assessed on pictorial, 5-point Likert scales adapted from IAPS (Lang et al., 2008). To assess these constructs, children were asked “On a scale from 1 to 5, with 1 being very sad and 5 being very happy, can you tell me how sad or happy the picture makes you feel?” and “On a scale from 1 to 5, with 1 being very bored and 5 being very excited, can you tell me how bored or excited the picture makes you feel?” Children rated a total of 180 images (Table 1). Images were grouped into six categories (large portions of high-ED foods, small portions of high-ED foods, large portions of low-ED foods, small portions of low-ED foods, large office supplies, and small office supplies). Each category of images was randomly divided into blocks of five images each. The presentation order of the blocks was counterbalanced within the questionnaire, but the order of the images within each block was kept consistent across children. Mean scores of excitability and emotional valence were calculated and then recoded to be on a scale of 0–4 instead of 1–5 for model interpretability. The questionnaire was self-paced, with children taking anywhere from 22 to 52 min to complete the ratings.

To confirm that the perceived sizes of the foods and objects were visually different between small and large amounts, two trained research assistants measured the items as they were projected on the computer monitor (Dell p1911 monitor 19 in. wide, 1,440 × 900 pixel resolution, Round Rock, TX, United States). Research assistants measured the tallest part (height) and widest part (width) of each pictured food and office supply using a ruler and then recorded the measurements in mm. Because different viewers may identify different boundaries for maximum height and width of amorphous foods (e.g., macaroni and cheese, and soup) and office supplies (e.g., rubber bands and paper clips), all measurements were compared and then remeasured if they differed by 5% or more on either dimension. Upon reaching inter-rater agreement for all foods, the two measurements were averaged to determine the measured size of foods/objects within the images.

### Data Analysis

All data analyses were conducted using SAS (version 9.4; SAS Institute, Inc., Cary, NC). Three-way analyses of variance (ANOVAs) using proc glm were conducted to determine whether image characteristics, specifically heights and widths, differed by version of the image set (1 or 2), item category (high-ED foods, low-ED foods, or office supplies), or amount (small or large). Because different children rated Image Set 1 and Image Set 2, differences in demographic and anthropometric characteristics were tested between cohorts. Independent sample *t*-tests were used to evaluate differences in continuous variables, and chi-square statistic was used for categorical variables. Outcomes of interest included mean emotional valence, mean excitability, and recognition, expressed as the percent of images correctly identified. For the primary aim, mixed linear models (proc mixed) with repeated measures were used with all images and fixed factors included image category (high-ED foods, low-ED foods, and office supplies), amount (smaller and larger), and image set (1 and 2). For secondary aims, only food images were included in the model; and fixed factors were image condition (high-ED and low-ED), amount (small and large), set (1 and 2), and taste/flavor characteristics (savory and sweet). All two-way interactions were tested and then removed from the final model if not significant. Three- and four-way interactions were not tested owing to the small sample size. A covariance structure of compound symmetry was used in all models. Additionally, potential covariates (e.g., fullness, child age, child sex, and child BMI *z*-score) were tested, and significant interactions between fixed factors and covariates as well as covariate main effects were kept in the model. For outcomes with significant effects, the Tukey–Kramer method was used to adjust significance levels for multiple pairwise comparison between least squares means. Analysis of covariance was used to further investigate significant interactions between factors and continuous covariates. There were no missing data, and all excitability, emotional valence, and familiarity mean scores were plausible; thus, data imputation and removal of outliers were not performed. Significance level was set a *p* < 0.05; and raw means ± standard deviations of participant characteristics and modelled means ± standard deviations for all other outcomes are reported.

## Results

### Image Properties

As shown in Table 2, there were no differences in width or height of the items by image set. As expected, large foods/office supplies were wider than small foods/office supplies (9.76 ± 2.70 vs. 5.90 ± 2.18 cm; *p* < 0.0001). Width of the pictured items differed by item category (*p* < 0.05), with high-ED foods measuring wider than office supplies (8.31 ± 2.75 vs. 7.48 ± 3.29 cm; *p* < 0.01). The high-ED foods were also marginally wider than the low-ED foods (8.31 ± 2.75 vs. 7.76 ± 3.21 cm; *p* = 0.06). Widths of low-ED foods and office supplies did not differ (7.48 ± 3.29 vs. 7.48 ± 3.29 cm; *p* = 0.318). For the height of the pictured items, the larger amounts were taller than the small amounts (6.84 ± 1.86 vs. 4.88 ± 1.49 cm; *p* < 0.0001). There was a marginal effect of item category on image height (*p* = 0.094), driven by differences in height between low-ED foods and office supplies (6.01 ± 1.93 vs. 5.59 ± 2.13 cm; *p* < 0.05). Height of high-ED did not differ from that of low-ED foods (5.91 ± 1.77 vs. 6.01 ± 1.93 cm, *p* = 0.58) or of office supplies (5.91 ± 1.77 vs. 5.59 ± 2.13 cm, *p* = 0.14).

**TABLE 2:** Widths and heights (cm) of pictured items in a standardized image set of high-energy-dense foods, low-energy-dense foods, and office supplies depicted in small and large amounts.

	**High-ED foods**		**Low-ED foods**		**Office supplies**		**Three-way ANOVA^a^**								
	**Set 1**	**Set 2**	**Set 1**	**Set 2**	**Set 1**	**Set 2**	**Effect of category**			**Effect of image set**			**Effect of amount**		
	**Mean ± *SD***	**Mean ± *SD***	**Mean ± *SD***	**Mean ± *SD***	**Mean ± *SD***	**Mean ± *SD***	**df**	***F***	***p***	**df**	***F***	***p***	**df**	***F***	***p***
**Width of pictured item (cm)**															
Small	6.38 ± 2.00	6.37 ± 1.98	5.98 ± 1.82	6.01 ± 1.83	5.36 ± 2.74	5.24 ± 2.72	2	3.70	0.025	1	0.00	0.975	1	262.81	<0.0001
Large	10.23 ± 1.93	10.26 ± 1.96	9.55 ± 3.28	9.49 ± 3.50	9.60 ± 2.26	9.68 ± 2.25									
**Height of pictured item (cm)**															
Small	4.76 ± 1.03	4.76 ± 0.98	5.17 ± 1.55	5.19 ± 1.58	4.56 ± 1.74	4.55 ± 1.75	2	2.38	0.094	1	0.02	0.932	1	141.83	<0.0001
Large	7.02 ± 1.69	7.07 ± 1.60	6.84 ± 1.90	6.87 ± 1.97	6.62 ± 2.02	6.61 ± 2.00									

*ED, energy dense. ^a^ Interactions between category, image set, and amount were tested but were not significant and were removed from the models.*

In addition to image size, other image properties were calculated using Matlab scripts created by Blechert et al. (2014) (adapted from https://github.com/nabusch/foodpics_code). Table 3 includes mean results on red, green, and blue color values, as well as image intensity, complexity, and mean spectral power. Additional information on properties for each image and comparisons across all conditions can be found in Supplementary Material. There were no differences in image properties between versions (*p* > 0.05 for all properties). As expected, image characteristics differed between larger and smaller amounts, with larger food and non-food images showing greater values for red and blue color, complexity, and mean spectral power (*p-*values ranging from 0.04 to 0.0001). In both Image Set 1 and Image Set 2, large office supplies had greater image intensity than small office supplies (*p* = 0.03 for both versions). In Image Set 1, high-ED foods had more red color than low-ED foods (*p* < 0.05), but there were no differences in image property between high-ED and low-ED foods among images in Image Set 2 (*p* > 0.05 for all properties).

**TABLE 3:** Mean image properties assessed in Matlab for food and non-food images grouped by energy density, version, and amount.

	**Image property**					
	**Image Set 1**			**Image Set 2**		
	**High-ED foods**	**Low-ED foods**	**Office supplies**	**High-ED foods**	**Low-ED foods**	**Office supplies**
**Red**						
Small	0.32 ± 0.01	0.35 ± 0.01	0.34 ± 0.01	0.36 ± 0.01	0.35 ± 0.01	0.34 ± 0.01
Large	0.37 ± 0.03	0.36 ± 0.02	0.35 ± 0.02	0.37 ± 0.01	0.36 ± 0.02	0.35 ± 0.02
**Green**						
Small	0.37 ± 0.004	0.33 ± 0.01	0.34 ± 0.01	0.34 ± 0.003	0.33 ± 0.01	0.34 ± 0.01
Large	0.34 ± 0.01	0.34 ± 0.01	0.34 ± 0.01	0.34 ± 0.01	0.34 ± 0.01	0.34 ± 0.01
**Blue**						
Small	0.31 ± 0.01	0.31 ± 0.01	0.32 ± 0.01	0.31 ± 0.01	0.31 ± 0.01	0.32 ± 0.02
Large	0.30 ± 0.02	0.30 ± 0.01	0.31 ± 0.01	0.30 ± 0.02	0.30 ± 0.01	0.31 ± 0.02
**Intensity**						
Small	144.08 ± 12.56	143.75 ± 16.16	142.91 ± 8.20	144.21 ± 12.55	143.14 ± 15.84	143.14 ± 8.22
Large	145.01 ± 12.05	150.10 ± 14.72	148.81 ± 9.10	144.95 ± 12.04	149.69 ± 14.55	149.01 ± 8.97
**Complexity**						
Small	0.07 ± 0.01	0.07 ± 0.02	0.06 ± 0.01	0.07 ± 0.01	0.07 ± 0.01	0.06 ± 0.10
Large	0.11 ± 0.04	0.11 ± 0.04	0.09 ± 0.03	0.11 ± 0.04	0.11 ± 0.04	0.10 ± 0.03
**Spectral power**						
Small	14.73 ± 0.18	14.71 ± 0.25	14.59 ± 0.26	14.78 ± 0.21	14.77 ± 0.29	14.62 ± 0.29
Large	15.08 ± 0.29	14.98 ± 0.50	14.78 ± 0.33	15.09 ± 0.28	15.08 ± 0.47	14.81 ± 0.36

**ED, energy dense.**

Among both Image Sets 1 and 2, there were differences in image properties between food images and office supplies. In both versions, high-ED foods had greater red (*p* < 0.0001), complexity (*p* < 0.05), and spectral power (*p* < 0.0001), and lower blue (*p* < 0.0001) than office supplies.

In sum, the images showed expected differences in visual properties between large and small amounts but did not systematically differ between high-ED and low-ED foods, suggesting they are well matched for image properties between food conditions. However, there were differences in image color, complexity, and spectral power between food images and office supplies.

### Participant Characteristics

A total of fifty-nine 6- to 10-year-olds were enrolled, completed all study procedures, and were included in analyses. Thirty children (60.0% male) rated Image Set 1, and 29 children (58.6% male) rated Image Set 2. As shown in Table 4, the majority of children were not Hispanic nor Latino and were white or Caucasian. Participating children were from families with a relatively high socioeconomic status as evidenced by limited use of free- or reduced-priced school meals and Medicaid and moderately high household income. Child demographics and anthropometrics did not differ between children who rated Image Set 1 and children who rated Image Set 2.

**TABLE 4:** Characteristics of 6- to 10-year-old children who rated the emotional valence, excitability, and recognition of a standardized image set developed investigate the cognitive and neurobiological mechanisms of eating behaviors.

	**Image Set 1 (*n* = 30)**		**Image Set 2 (*n* = 29)**		***p*-value**
	**Mean ± *SD***	**Range**	**Mean ± *SD***	**Range**	
Child age (years)	8.3 ± 1.2	6.2–10.7	8.1 ± 1.1	6.1–10.4	0.77^a^
Sex-specific BMI-for-age percentile	67.7 ± 22.9	6.3–99.0	64.09 ± 24.5	7.6–99.3	0.72^a^
Sex-specific BMI-for-age *z*-score	0.60 ± 0.86	−1.53 to 2.32	0.51 ± 0.90	−1.43 to 2.47	0.81^a^
Fullness	55.1 ± 36.9	0–123	50.2 ± 37.1	0–143.0	0.98 ^a^
Parent age (years)	39.5 ± 4.6	34.1–48.9	40.4 ± 6.0	32.2–55.4	0.14 ^a^
Parent body mass index (kg/m^2^)^c^	29.0 ± 7.7	19.7–50.4	30.3 ± 8.5	18.9–52.9	0.61 ^a^

	***N* (%)**		***N* (%)**		***p*-value**

**Child sex**					
Male	18 (60.0%)		17 (58.6%)		0.91^b^
Female	12 (40.0%)		12 (42.4%)		
**Child weight status^d^**					
Normal weight	22 (73.3%)		22 (75.9%)		0.82^b^
Overweight/obese	8 (26.7%)		7 (24.1%)		
Infant feeding method					0.71^b^
Breast-fed	20 (66.7%)		22 (75.9%)		
Formula fed	9 (30.0%)		6 (20.7%)		
Prefer not to say	1 (3.3%)		1 (3.4%)		
Person primarily responsible for child feeding					0.19^b^
Myself	18 (60.0%)		17 (58.6%)		
My partner	1 (3.3%)		3 (10.3%)		
Both	11 (36.7%)		9 (31.0%)		
How frequently family eats out or gets takeout					0.60^b^
Once per month or less	5 (16.7%)		3 (10.3%)		
Twice a month	9 (30.0%)		9 (31.0%)		
Once a week	9 (30.0%)		11 (37.9%)		
Two times a week	5 (16.7%)		6 (20.7%)		
Three times a week	2 (6.7%)		0 (0%)		
**Use of free or reduced-priced meals**					
Full price	27 (90.0%)		21 (72.4%)		0.17^b^
Reduced price	1 (3.3%)		5 (17.2%)		
Free	2 (6.7%)		3 (10.3%)		
**Household income**					
Less than $20,000	0 (0%)		0 (0%)		0.58^b^
$20,000–35,999	1 (3.3%)		4 (13.8%)		
$36,000–50,999	1 (3.3%)		3 (10.3%)		
$51,000–75,999	8 (26.7%)		5 (17.2%)		
$76,000–100,000	4 (13.3%)		4 (13.8%)		
More than $100,000	15 (50.0%)		12 (41.4%)		
Missing	1 (3.3%)		1 (3.5%)		
**Medicaid use**					
No	24 (80.0%)		23 (79.3%)		0.95^b^
Yes	6 (20.0%)		6 (20.7%)		
**Child ethnicity**					
Not Hispanic or Latino	27 (90.0%)		29 (100%)		0.22^b^
Hispanic or Latino	2 (6.7%)		0 (0%)		
**Missing**	1 (3.3%)		0 (0%)		
Child race					
Asian	0 (0%)		2 (6.9%)		0.29^b^
Black/African American	2 (6.7%)		3 (10.3%)		
White/Caucasian	28 (93.3%)		24 (82.8%)		

*^a^ Differences between means of participants in Image Set 1 and Image Set 2 were evaluated by independent-sample t-tests. ^b^ Differences between the distributions on categorical variables between Image Set 1 and Image Set 2 were evaluated by chi-square statistic. ^c^ N = 28 for Image Set 1 and N = 28 for Image Set 2. ^d^ Child weight and height measurements were converted to body mass index (BMI) and converted to sex-specific BMI-for-age percentiles based on the Centers for Disease Control and Prevention (Cole et al., 2000).*

### Recognition, Excitability, and Emotional Valence

#### Relationship Between Image Ratings

The relationship between image recognition, excitability, and emotional valence is detailed in Table 5. There were positive associations between emotional valence and excitability for high-ED foods (*r* = 0.88, *p* < 0.01), low-ED foods (*r* = 0.80, *p* < 0.01), and office supplies (*r* = 0.87, *p* < 0.01). Recognition was not related to emotional valence or excitability for high-ED or low-ED foods (*p*>0.05 for both). Both emotional valence (*r* = 0.21, *p* < 0.05) and excitability (*r* = 0.22, *p* < 0.05) were moderately related to children’s recognition of office supplies.

**TABLE 5:** Pearson’s correlations between image ratings for recognition, emotional valence, and excitement.

**Image category**	**Recognition**	**Emotional valence**	**Excitement**
**High-ED foods**			
Recognition		−0.12	−0.05
Emotional valence	−0.12	–	0.88**
Excitement	−0.05	0.88**	–
**Low-ED foods**			
Recognition		0.14	0.11
Emotional valence	0.14	–	0.80**
Excitement	0.11	0.80**	–
**Office supplies**			
Recognition		0.21*	0.22*
Emotional Valence	0.21*	–	0.87**
Excitement	0.22*	0.87**	–

*p < 0.05; **p < 0.01.

#### Recognition

**FIGURE 3:**
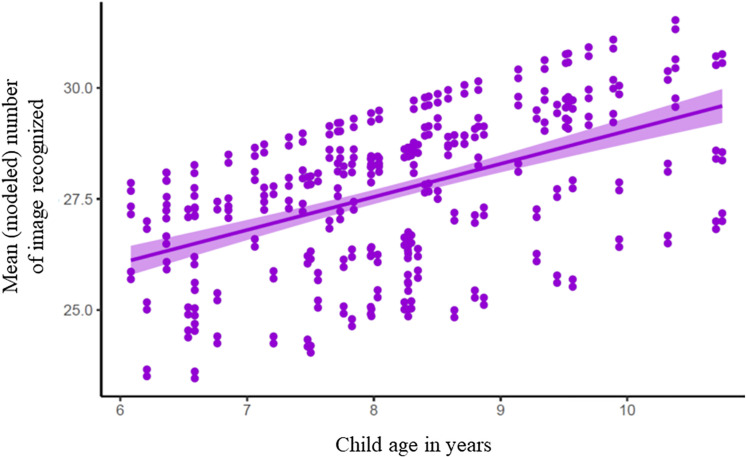
Accuracy of recognition of images. depicting high-ED. low-ED. and office supplies. increased with child age [*P* = 0.001; *F*(155) = 11.52. Cohen’s d = 0.44] and recognition increased by 2.7% (SE = 0.78) for each year increase in age in a sample of children ranging froni 6.08 to 10.75 years of age. Modeled slope and standard errors along with individual data points are values extracted from the model.

**FIGURE 4:**
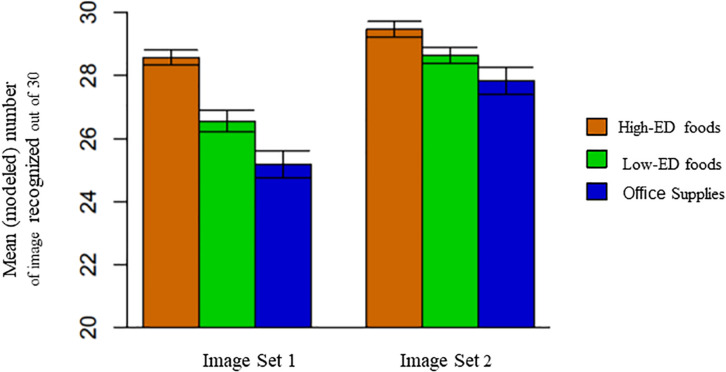
Children’s recognition of 30 high-ED food images. 30 low-ED food images. and 30 office supply images between Image Set 1 and Image Set 2 of the images. Improvements in image recognition were attributable to improvements made in recognition of low-ED foods and office supplies not high-ED foods [version × image category interaction term: *P* < 0.000 1: *F*(2gg) = 12.53].

Children’s recognition and correct identification of the images did not differ between the small and large amounts of the pictured items (92.7 ± 7.2 vs. 92.1 ± 7.2%; *p* = 0.223) but did vary by the age of the child at testing [*F*_(1, 55)_ = 11.52; *p* ≤ 0.001] as shown in Figure 3. On average, recognition increased by 2.7% (SE = 0.78) for each year increase in age in a sample of children ranging from 6.08 to 10.75 years old. As initial testing with Image Set 1 showed lower recognition of 10 images (two high-ED foods, four low-ED foods, and four office supplies), replacement of these images with more familiar items in Image Set 2 improved recognition of low-ED and office supplies but not high-ED foods (version × image category interaction term [*F*_(2, 288)_ = 12.53; *p* < 0.0001]. As shown in Figure 4, recognition improved from Image Set 1 to Image Set 2 for low-ED food images (88.3 ± 10.5 vs. 95.6 ± 10.6%; *t* value = −3.73; *p* < 0.0001) and office supplies (83.7 ± 10.5 vs. 93.0 ± 10.6%; *t* value = −4.81; *p* < 0.0001), but not high-ED foods (95.0 ± 10.5 vs. 98.4 ± 10.6%; *t* value = −1.74; *p* = 0.50), which were already highly recognized in the first image set.

Planned comparisons were used to examine recognition by image category to see whether there were differences in Image Set 2. Children had better recognition of high-ED foods than low-ED foods (98.4 ± 10.6 vs. 95.6 ± 10.6%; *t* value = 3.25; *p* < 0.005) and of high-ED foods relative to office supplies (98.4 ± 10.6 vs. 93.0 ± 10.6%; *t* value = 6.23; *p* < 0.0001). Recognition of low-ED foods was higher than recognition of office supplies (95.6 ± 10.6 vs. 93.0 ± 10.6%; *t* value = 2.98; *p* < 0.05). The child’s perceived fullness influenced the relationship between category of item pictured and recognition accuracy [*F*_(2, 288)_ = 4.78; *p* < 0.01], however, this relationship was no longer significant after removal of an outlier (a 6.08-year-old male) whose recognition score was 2 standard deviations below the mean. All other results remained the same.

#### Excitability

**FIGURE 5:**
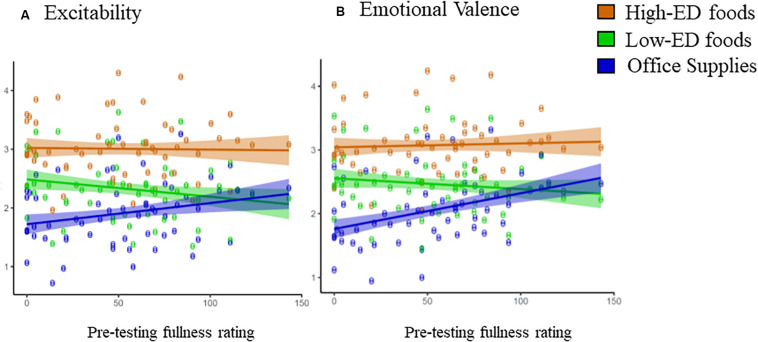
The effect of an interaction between category of image (e.g., high-ED, low-ED, and office images) and fullness score on ratings of image excitability and emotional valence-A. The slopes between excitability and fullness differed by the category of item pictured [*p* = 0.002; *F*(290) = 634]. The slope for office supplies was greater than the slope for high-ED and low-ED foods; however, the slope for low-ED foods did not differ from the slope for high-ED food& Ratings of excitability of low-ED foods and office supplies were significantly different at lower levels of fullness, but were not significantly different at higher levels of fullness- B. The slopes between emotional valence and fullness differed by the category of item pictured [*p* = 0M004; *F*(2294) = 7.92]. The slope for office supplies was greater than the slope for high-ED foods and office supplies, however, the slope for low-ED foods did not differ from the slope for high-ED foods. Ratings of emotional valence of low-ED foods and office supplies were significantly different at lower levels of fullness, but were not significantly different at higher levels of fullness.

Excitability ratings of the images did not differ between Image Sets 1 and 2 [2.44 ± 0.8 vs. 2.39 ± 0.9; *F*_(1, 56)_ = 0.09; *p* = 0.77] or by amount of the item pictured [2.43 ± 0.6 vs. 2.40 ± 0.6; *F*_(1, 290)_ = 0.32; *p* = 0.57]. Independent of amount and version, there was a main effect of category on the child’s excitability rating [*F*_(2, 390__)_ = 60.90; *p* < 0.0001]. Children had higher excitability ratings for high-ED relative to low-ED foods (3.01 ± 0.68 vs. 2.33 ± 0.68; *t*-value = 10.04; *p* < 0.0001) and office supplies (3.01 ± 0.68 vs. 1.91 ± 0.68; *t*-value = 16.26; *p* < 0.0001). Excitability was also higher for low-ED foods than office supplies (2.33 ± 0.68 vs. 1.91 ± 0.68; *t* value = 6.22; *p* < 0.0001). However, the effect of category differed depending on the child’s reported fullness level prior to testing [*F*_(2, 290)_ = 6.34; *p* < 0.01]. As depicted in Figure 5A, the slope for office supplies was greater than the slopes for both high-ED foods (*p* < 0.05) and low-ED foods (*p* < 0.001). There were no differences between the slopes for low-ED and high-ED foods (*p* = 0.15). The negative slopes for both high- and low-ED foods indicate that children’s excitement increased as fullness levels decreased, suggesting that children found food images more exciting when they were hungry. On the other hand, the positive slope for office supplies suggests that excitement for these items was lowest when the children were hungry (i.e., low fullness levels). As a result of the differences in slopes, at the lowest level of fullness (0 mm), children gave higher excitability ratings to low-ED foods relative to office supplies, but at the highest level of fullness (144 mm), excitability for low-ED foods relative to office supplies did not differ.

#### Emotional Valence

Emotional valence ratings did not differ between Image Sets 1 and 2 (*p* = 0.98) or between small vs. large amounts (*p* = 0.54). As was the case with excitability, there was a main effect of category on the child’s emotional valence rating [*F*_(2, 390)_ = 60.90; *p* < 0.0001]. Children had higher emotional valence for high-ED relative to low-ED foods (3.07 ± 0.63 vs. 2.47 ± 0.63; *t* value = 8.90; *p* < 0.0001) and office supplies (3.07 ± 0.63 vs. 2.06 ± 0.63; *t*-value = 14.90; *p* < 0.0001). Emotional valence was also higher for low-ED foods than office supplies (2.47 ± 0.63 vs. 2.06 ± 0.63; *t*-value = 6.01; *p* < 0.0001). Similarly to excitability, emotional valence rating was also dependent on the child’s reported fullness level prior to testing [*F*_(2, 290)_ = 7.92; *p* < 0.0001]. As depicted in Figure 5B, the slope for office supplies was greater than the slopes for high-ED foods (*p* < 0.01) and low-ED foods (*p* ≤ 0.0001). The slopes for low-ED and high-ED foods were not different (*p* = 0.22). The slope of the relationship between emotional valence and fullness was significantly different from zero for office supplies (*p* < 0.05) but not high-ED foods (*p* = 0.79) or low-ED foods (*p* = 0.45). Thus, for each 1-mm increase on the fullness scale, emotional valence of office supplies increased by 0.006 points. As a result of the differences in slopes, at the lowest level of fullness (0 mm), children gave higher emotional valence ratings to low-ED foods relative to office supplies, but at the highest level of fullness (144 mm), emotional valence for low-ED foods relative to office supplies did not differ.

#### Recognition of Savory vs. Sweet Foods

Because the image set was approximately balanced with respect to major taste characteristic (sweet vs. savory), exploratory analyses were conducted to determine whether recognition, emotional valence, and excitability differed among image subgroups. There was no interaction between taste characteristics (sweet vs. savory) and amount (large vs. small) on image recognition (*p* = 0.21). However, there was an interaction between taste and item category [*F*_(1, 408)_ = 65.53; *p* < 0.0001]. For savory foods, recognition of high-ED foods was higher than recognition of low-ED foods (97.7 ± 7.2 vs. 88.8 ± 7.2%; *t* value = −12.22; *p* < 0.0001), whereas recognition did not differ between high-ED and low-ED foods that were sweet (95.7 ± 7.2 vs. 95.2 ± 7.2%; *p* = 0.87). For high-ED foods, recognition was higher for savory foods than sweet foods (97.7 ± 7.2 vs. 95.7 ± 7.2%; *t* value = 2.71; *p* < 0.05). On the other hand, for low-ED foods, recognition was higher for sweet than savory foods (95.2 ± 7.2 vs. 88.8 ± 7.2%; *t* value = −8.73; *p* < 0.0001). Lastly, there was an interaction between taste characteristic and image set [*F*_(1, 408)_ = 4.19; *p* < 0.05], showing that improvements in recognition between image sets were attributable to increased recognition of savory, but not sweet foods. However, this interaction was no longer significant following removal of the child who was an outlier. All other results, however, remained the same.

#### Excitability of Savory vs. Sweet Foods

There were no interactions between taste characteristics (savory vs. sweet) and image set, amount, or image category (all *p* > 0.14). Independent of image set and amount, taste characteristics of the images were related to children’s excitability ratings; scores were higher for sweet foods than for savory foods [3.01 ± 0.63 vs. 2.33 ± 0.63; *F*_(1, 410)_ = 215.78; *p* < 0.0001].

#### Emotional Valence of Savory vs. Sweet Foods

Interactions between taste characteristics (savory vs. sweet) and image set, amount, or image category were not statistically significant predictors of emotional valence scores (all *p*’s > 0.30). Independent of image set and amount, taste characteristics of the food images were related to emotional valence: scores were higher for sweet foods than for savory foods [3.08 ± 0.58 vs. 2.46 ± 0.58; *F*_(1, 410)_ = 185.33; *p* < 0.0001].

## Discussion

The present paper describes the development and preliminary testing of a set of standardized images for studying food-cue reactivity in children. This image set advances the literature and adds to a growing library of freely available, standardized pictures for studying individual variation in human eating behaviors (Foroni et al., 2013; Blechert et al., 2014, 2019; Miccoli et al., 2014; Charbonnier et al., 2016; King et al., 2018). Across the three categories of images (i.e., high-ED foods, low-ED foods, and office supplies), we found high recognition (i.e., over 90%), and, as expected, high-ED foods were more exciting and emotionally salient than both low-ED foods and office supplies. However, there were no differences in recognition, excitability, and emotional valence between large and small portions/amounts. In addition, high- and low-ED foods were well matched for common visual properties, like image color and complexity. To help characterize novel risk factors for early disordered eating and obesity, these images can be used to investigate individual differences in food-cue responsivity among pediatric cohorts.

To produce valid and reliable physiological or psychological responses, images must first and foremost be recognizable. Prior standardized image sets developed primarily for use with adults have shown lower recognition when tested in pediatric samples (Charbonnier et al., 2016). Therefore, our primary goal was to select foods and control stimuli that were familiar to children. To achieve this, we relied on population-level data to provide information on the most common foods eaten by preadolescent children in the United States (Smiciklas-Wright et al., 2003). Office supplies were selected as the non-food control because they could resemble the size, shape, and color of many foods and because children are often exposed to these items at school. Additionally, we tested the images on two independent samples of children and replaced foods/office supplies that were poorly recognized. These changes were successful, and the final version had improved recognition, with minimal differences between food and non-food conditions. Children’s recognition of images increased with age, a finding that likely was associated with increased exposure to a variety of foods and office supplies. As shown in the literature, dietary variety increases as children grow older (Fernandez et al., 2016) and older children would have spent more time in school where they would have been exposed to a range of office supplies. In regard to recognition of pictured foods and items, investigators who plan to use these images should consider limiting the age range to 7 years and older to increase the likelihood that participants will recognize the images. In pediatric populations, adjusting for child age in statistical analyses is also recommended.

As hypothesized, children gave higher ratings of excitability and emotional valence to high-ED than low-ED foods, providing evidence of construct validity for the images. Similar observations were found in the image set developed by Miccoli et al. (2014) which was tested with adolescents from Spain. In addition, ratings for excitability and emotional valence were highly correlated with one another, which is consistent with other reports in children and adolescents who have used IAPS rating scales to evaluate pictures (Mathias et al., 2012; Miccoli et al., 2014). Prior studies have found that children report the highest liking for energy-dense foods that are high in sugar and fat, whereas vegetables tend to be the least liked (Cooke and Wardle, 2007). From a young age, children learn to like higher fat foods, many of which are also high in energy density, because they provide a source of energy and they are often presented in positive social contexts (e.g., cupcakes served at a birthday party) (Birch, 1992). Also confirming our expectations based on children’s increased liking of sweet foods (Birch, 1979) we found that images of sweet foods were rated as more exciting and emotionally salient than images of savory foods. Overall, the results suggest that the images are evoking an expected pattern of affective responses from children.

Although we found expected differences in children’s ratings of the food images based on energy density, there were no differences in affective evaluations between larger and smaller portions. Prior research has shown that increasing the portion size of foods and beverages has a robust effect on children’s intake (Fisher et al., 2007; Mathias et al., 2012; Kling et al., 2016; Smethers et al., 2019). Children’s food preferences also differ by the type and amount of food served. For example, children prefer larger portions of higher-ED, palatable foods and smaller portions of vegetables (Colapinto et al., 2007). Children’s responses to differences in portion size, however, may not be captured under the constructs of emotional valence and excitability. Measuring other constructs, such as liking or wanting, may reveal differences in children’s responses to images depicting smaller and larger portions of food. Furthermore, although the amounts for food and office supplies were chosen to depict obvious differences in portion, children’s ability to discriminate between the smaller and larger amounts was not formally measured. Lastly, the smaller portion size aligns with standard portions from a US representative dataset (Smiciklas-Wright et al., 2003) but it is unknown how these portions relate to what the participants in our cohort typically consume. On the basis of these findings, investigators who are interested in understanding individual differences in children’s emotional valence and excitability to food images could select the level of portion size most appropriate to their population.

The images developed offer several strengths. To our knowledge, there have been no prior image sets developed specifically for use in pediatric studies that include foods commonly consumed by children in the United States. Second, the foods are pictured in both standard and “large” portion sizes. For this reason, the image set is ideal for testing questions related to both the individual and combined effects of energy density and portion size on psychological responses. An additional strength of this image set is that foods were selected in a systematic fashion, based on population data of foods children in the United States commonly consumed (Smiciklas-Wright et al., 2003). Whereas several other food databases consist of images found on the internet (Foroni et al., 2013; Blechert et al., 2014, 2019; Miccoli et al., 2014) our images were photographed in the laboratory and are highly consistent and reasonably well matched for properties, like color, intensity, and complexity. Matching of image visual properties is especially important for neuroimaging research, as differences in these characteristics can evoke different neural response patterns. Furthermore, details on the image characteristics are included in Supplementary Material to help researchers make informed decisions about which images to include in future studies.

There are also several areas in which the current image set can be improved upon in future iterations. Because we developed the images on the basis of input from data collected on the diets of US children, the results are unlikely to be generalizable to other cultures and may not be representative of more diverse samples within the United States. In addition, our sample size was small and homogeneous in terms of ethnicity and socioeconomic status. Testing the images in a larger, more diverse cohort is necessary to understand the generalizability of our findings. We would suggest that if investigators use these images outside of the United States and/or in more culturally heterogeneous populations, they first conduct pilot testing with their sample to ensure the images are salient and familiar. Moreover, compared with other image sets, the current dataset is small and limited in its variety of food types and cuisines. A goal for the future will be to expand upon the images by including a greater overall number of foods that are representative of more diverse cultural eating habits. Although our food images were well matched for visual properties, we found differences between food images and office supplies for image color, size, and complexity. Although we attempted to balance the use of colors across the groups (i.e., high-ED foods, low-ED foods, and office supplies), we were not able to fully match the shapes and sizes of the office supplies to those of the foods. However, this raises a point for discussion in regard to how closely non-food images should resemble real foods. Some prior studies have attempted to match food and non-food images for image size, color, and complexity (Deckersbach et al., 2014). In some cases, this can impede recognition of the non-food objects and may cause participants to mistake them for actual foods. In the initial development of our image set, we removed several office supplies that too closely resembled food items (e.g., yellow Post-it Notes that looked like American cheese) to avoid inadvertently evoking affectual responses to those stimuli. Finally, we do not have information on other food attributes, such as liking, taste, and health. With 180 total images, we were limited in the number of attributes we could feasibly measure with 6- to 10-year-old children. Future studies will be needed to collect data on other perceptual qualities of the images in this dataset.

## Conclusion

We developed a food image database tailored for the study of the neurocognitive mechanisms of eating behavior in children. The key strengths of this image set are the high familiarity and standardization of the images and the inclusion of commonly eaten foods at two levels of energy density and two levels of portion size. The set also includes control images of office supplies that vary by the amount pictured. The use of this image set by investigators will improve the ability to replicate research findings on the individual differences in food-cue responsivity among children.

## Data Availability Statement

The raw data supporting the conclusions of this article will be made available by the authors, without undue reservation, to any qualified researcher.

## Ethics Statement

The studies involving human participants were reviewed and approved by the Pennsylvania State University Internal Review Board. Written informed consent to participate in this study was provided by the participants’ legal guardian/next of kin.

## Author Contributions

SK was responsible for data analysis and preparation of initial drafts. MR was responsible for image design and development and data collection. AP was responsible for data analysis and presentation and manuscript editing. HG, CG, ER, and SW was responsible for experimental design and editing. BR was responsible for experimental design, image development, and editing. KK was responsible for project conception, image design and development, data management, manuscript preparation, and editing. All authors contributed to the article and approved the submitted version.

## Conflict of Interest

The authors declare that the research was conducted in the absence of any commercial or financial relationships that could be construed as a potential conflict of interest.
